# Comparison of Non-Invasive and Invasive Diagnostic Methods for Coronary Artery Disease: Single-Centre Data

**DOI:** 10.3390/medicina62030452

**Published:** 2026-02-27

**Authors:** Tautvydas Fabijonavičius, Lina Gastilavičiūtė, Gerda Falkauskaitė, Martynas Jurėnas, Ramūnas Unikas, Mindaugas Barauskas

**Affiliations:** 1 Faculty of Medicine, Medical Academy, Lithuanian University of Health Sciences, 44307 Kaunas, Lithuania; lina.gastilaviciute@stud.lsmu.lt (L.G.); gerda.falkauskaite@stud.lsmu.lt (G.F.); 2 Department of Cardiology, Medical Academy, Lithuanian University of Health Sciences, 44307 Kaunas, Lithuania; martynas.jurenas@lsmu.lt (M.J.); ramunas.unikas@lsmu.lt (R.U.); mindaugas.barauskas@lsmu.lt (M.B.); 3 Heart Centre, Kauno Klinikos, Hospital of Lithuanian University of Health Sciences, 50161 Kaunas, Lithuania

**Keywords:** coronary computed tomography angiography, invasive coronary angiography, diagnostic agreement, myocardial perfusion scintigraphy, coronary artery disease, coronary artery stenosis

## Abstract

*Background and Objectives:* Coronary computed tomography angiography (CCTA) is widely used in the diagnostic evaluation of suspected stable coronary artery disease; however, its agreement with invasive coronary angiography (ICA) remains inconsistent across different levels of analysis. The aim of this study was to evaluate the agreement between CCTA and ICA and to identify the factors associated with discrepancies. *Materials and Methods:* A single-centre retrospective analysis of 500 patients was performed. All patients underwent CCTA within one year prior to ICA. Coronary stenoses were evaluated at the 11-segment coronary artery, vessel, and patient levels using a ≥50% cut-off. Diagnostic agreement was assessed using the kappa coefficient, while diagnostic performance was evaluated in terms of sensitivity, specificity, positive predictive value, and negative predictive value. Factors associated with discrepancies were evaluated using a logistic regression model. *Results:* At the segment level, agreement between CCTA and ICA was low to moderate across 11 coronary segments (κ = 0.108–0.461). At the patient level, CCTA identified ≥50% coronary stenosis more frequently than ICA (86.2% vs. 59.4%, *p* < 0.001), demonstrating high sensitivity (91.3%) but low specificity (21.2%). Diagnostic discrepancies were associated with higher coronary calcium burden, and in multivariable analysis, body mass index > 25 kg/m^2^, age < 68 years, and multiple comorbidities were independently associated with discordant findings. *Conclusions:* At the patient level, CCTA demonstrates high sensitivity and represents an appropriate non-invasive method for patient selection for further diagnostic evaluation. However, agreement between CCTA and invasive coronary angiography remains limited at the segment and vessel levels. Diagnostic discrepancies were significantly associated with coronary artery calcification and higher body mass index (BMI), which should be taken into consideration when interpreting CCTA findings.

## 1. Introduction

Coronary artery disease (CAD) remains one of the leading causes of morbidity and mortality worldwide, emphasising the need for accurate and timely diagnostic methods [1]. Invasive coronary angiography (ICA) is the reference standard for assessing coronary artery anatomy, offering high resolution and enabling immediate intervention. However, the invasive character of ICA and associated risks, including vascular complications and radiation exposure, underscore the need for less invasive alternatives [2,3]. Non-invasive diagnostic strategies, particularly coronary computed tomography angiography (CCTA), have emerged as one of the first-line modalities for the evaluation of suspected stable CAD, recommended by current guidelines for patients with low-to-intermediate pretest probability [2,3]. Despite its widespread adoption, discrepancies between CCTA and ICA findings are frequently observed, especially when coronary stenosis severity is categorised using threshold-based definitions [4]. Moreover, discordance between anatomical imaging and functional ischemia assessment, evaluated by myocardial perfusion scintigraphy (MPS), further complicates clinical decision-making regarding the need for invasive evaluation and revascularisation.

These discrepancies highlight the need for detailed evaluation of CCTA diagnostic performance. Variations in CCTA accuracy across the coronary tree, influenced by factors such as vessel size, calcification, and patient characteristics, necessitate a detailed analysis. The main aim of this study was to evaluate the concordance between non-invasive (CCTA and MPS) and invasive (ICA) test results, to identify factors determining the discordance between these methods, and to assess their clinical significance in decision-making regarding invasive treatment. For this reason, only patients who underwent invasive coronary angiography were included in the analysis, allowing direct comparison of non-invasive and invasive examination results.

## 2. Materials and Methods

### 2.1. Study Design and Population

A single-centre retrospective analysis was conducted at the Department of Cardiology, Hospital of Lithuanian University of Health Sciences Kaunas Clinics, Kaunas, Lithuania. The analysis included adult patients who underwent invasive coronary angiography (ICA) between 1 January 2025, and 1 July 2025. All included patients were required to have undergone coronary computed tomography angiography (CCTA) within one year prior to ICA. Myocardial perfusion scintigraphy (MPS) data, when available, were collected and analysed if performed within the same one-year period. The study cohort consisted exclusively of outpatients evaluated for stable coronary artery disease; in our dataset, there were no cases in which a patient underwent CCTA and subsequently experienced an acute coronary syndrome while awaiting invasive coronary angiography. As only patients referred for invasive coronary angiography were included, the study population represented a clinically selected cohort with suspected significant coronary artery disease. After exclusion of patients with incomplete data or those not meeting eligibility criteria, a total of 500 consecutive patients with complete clinical and imaging datasets who met all predefined inclusion and exclusion criteria were included in the final analysis. All patients provided general written consent at the time of first registration at Kaunas Clinics, allowing the use of anonymised medical data for scientific purposes. Heart failure was defined as a clinical diagnosis based on symptoms and signs, whereas left ventricular failure was defined as isolated LV dysfunction, most often confirmed by echocardiography. These categories were not mutually exclusive.

### 2.2. Indications for Imaging and Diagnostic Pathway

Invasive coronary angiography was performed in patients in whom CCTA revealed anatomically significant coronary artery stenosis (≥50%) and/or who had clinical indications for further invasive evaluation. Myocardial perfusion scintigraphy was performed only in patients in whom further assessment of the functional significance of coronary stenosis was recommended based on CCTA findings. In these cases, MPS was performed prior to ICA as an intermediate functional diagnostic test.

### 2.3. Exclusion Criteria

Exclusion criteria included incomplete clinical or imaging data and absence of consent for the use of data. Additionally, patients with anatomical abnormalities, advanced or end-stage cardiac disease, metastatic malignancy, or other conditions interfering with the reliable interpretation of coronary artery imaging were not included.

### 2.4. Imaging Protocols and Reference Standards

CCTA was performed using a 320-slice computed tomography scanner (Aquilion ONE TSX-305A, Canon Medical Systems, Tochigi, Japan), using an electrocardiographically synchronised acquisition protocol. CCTA was used exclusively for anatomical assessment of coronary arteries. Invasive coronary angiography was considered the reference standard for the evaluation of coronary artery stenosis severity. The functional significance of coronary stenosis during ICA was assessed using the instantaneous wave-free ratio (iFR); stenosis was considered hemodynamically significant when iFR ≤ 0.89. Physiological assessment using iFR was performed as part of routine clinical practice at the discretion of the interventional cardiologist, based on clinical presentation and angiographic lesion characteristics. No dedicated prospective collection of iFR data had been planned; therefore, only patients with available iFR measurements were included (*n* = 31). Myocardial perfusion scintigraphy was performed using single-photon emission computed tomography (SPECT) with 99mTc-MIBI as the radiopharmaceutical agent. All imaging studies were interpreted by experienced specialists in routine clinical practice. Coronary dominance (right, left, or balanced) was recorded retrospectively from the final clinical CCTA and ICA reports, and no additional reclassification was performed by the study investigators. Due to the retrospective design and complete anonymisation of data, individual readers could not be identified; therefore, analyses were based on final clinical reports.

### 2.5. Coronary Artery Segmentation and Stenosis Classification

CCTA results were assessed according to the American Heart Association (AHA) 17-segment model, while ICA findings were evaluated using a 20-segment scheme. To ensure comparability between modalities, coronary artery segments were harmonised into 11 analytical segments. Segment-level analyses included the proximal (prox), mid (mid), and distal (dist) segments of the right coronary artery (RCA), left anterior descending artery (LAD), and left circumflex artery (LCX), as well as the left main coronary artery (LM) and the intermediate branch (IM). In each segment, stenosis severity was classified using a six-grade scale: 0%, <50%, 50–69%, 70–89%, 90–99%, and 100%. Additionally, a binary classification was applied, categorising stenosis as <50% or ≥50%.

### 2.6. Vessel-Level and Patient-Level Definitions

For vessel-level analysis, segment-level data were aggregated into three major coronary arteries: RCA (RCA_prox, RCA_mid, RCA_dist), LAD (LAD_prox, LAD_mid, LAD_dist), and LCX (LCX_prox, LCX_mid, LCX_dist). For patient-level analysis, significant coronary artery disease was defined as the presence of at least one coronary segment with stenosis ≥50%.

### 2.7. Statistical Analysis

Statistical analyses were performed using IBM SPSS Statistics version 30.0 (IBM Corp., Armonk, NY, USA). Quantitative variables are presented as the mean ± standard deviation or median with interquartile range, depending on distribution. Categorical variables are expressed as absolute numbers and percentages. Group comparisons were performed using the χ^2^ test or Fisher’s exact test for categorical variables and Student’s *t*-test or Mann–Whitney U test for continuous variables, as appropriate. Diagnostic agreement between CCTA and ICA was assessed using Cohen’s kappa (κ) coefficient, applying both linear and quadratic weighting. Diagnostic performance was evaluated by calculating sensitivity, specificity, positive predictive value (PPV), negative predictive value (NPV), and overall accuracy. Differences between diagnostic methods at the patient level were assessed using the McNemar test. Diagnostic discriminatory ability was evaluated using receiver operating characteristic (ROC) curve analysis, with the area under the curve (AUC) and 95% confidence intervals reported. ROC analysis evaluating the association between CCTA findings and invasive treatment was performed at the patient level. Factors associated with discrepancies between CCTA and ICA findings were analysed using binary logistic regression. Variables with *p* < 0.05 in univariable analysis were considered eligible for inclusion in the multivariable logistic regression model. Age was dichotomised according to the median value of the study population (68 years) for inclusion in the multivariable model. Coronary calcium burden (Agatston score) was analysed separately in artery-specific discrepancy analyses. It was not included in the final patient-level multivariable model because Agatston data were not available for all patients and inclusion would have reduced the effective sample size and increased the risk of model overfitting. The results are presented as odds ratios (ORs) with 95% confidence intervals. A *p*-value < 0.05 was considered statistically significant.

### 2.8. Literature Search

A structured, non-systematic literature search was performed to identify relevant studies supporting the background, interpretation, and discussion of the present findings. PubMed/MEDLINE, Web of Science and reference lists of key articles and guidelines were screened to identify additional relevant publications. The literature search primarily focused on studies published between January 2000 and December 2025, reflecting the era of modern CCTA. Earlier foundational studies were additionally considered where relevant to provide anatomical or conceptual background. Search terms included combinations of keywords related to CCTA, ICA, diagnostic accuracy (including vessel-based analysis), image quality/artifacts, coronary calcification, and functional assessment (FFR/iFR and CT-FFR). Eligible studies were peer-reviewed articles, original research articles or major reviews or guidelines, studies conducted in humans and published in English. The exclusion criteria were case reports, studies with very small samples, outdated technical protocols, and unclear diagnostic criteria. This literature search was intended to support the background and interpretation of the study findings and was not designed as a formal systematic review or meta-analysis.

## 3. Results

### 3.1. Study Population

The study included 500 patients: 249 (49.8%) men and 251 (50.2%) women. The mean age of the study population was 67.3 ± 9.4 years. Women (70.0 [64.0–75.0] years) were statistically significantly (*p* < 0.001) older than men (65.0 [59.0–73.0] years). The median body mass index (BMI) of the study population was 29.3 [26.0–33.2] kg/m^2^. Between men (28.7 [25.4–31.8] kg/m^2^) and women (29.8 [26.5–33.7] kg/m^2^), the difference was not statistically significant (*p* = 0.085). In total, 494 patients had at least one of the conditions listed in Table 1. The most frequent were arterial hypertension (94.8%), left ventricular failure (79.6%), and rhythm or conduction disorders (21.8%).

### 3.2. Segment-Wise Analysis of CCTA and ICA Results

#### 3.2.1. Agreement Analysis Based on Kappa Coefficients

According to the six-grade classification, the weighted kappa (κ) coefficient ranged from 0.108 to 0.436, and according to the two-grade classification, from 0.110 to 0.461. In both classifications, the highest agreement was observed in RCA_prox and the lowest κ values were observed in LCX_dist, IM and LM. Detailed segment-level results are presented in Table 2. The paired Wilcoxon signed-rank testing revealed statistically significant differences between CCTA-derived and ICA-derived six-grade stenosis classifications in most coronary segments (*p* < 0.05), while no significant differences were observed in the RCA_dist and IM.

#### 3.2.2. Diagnostic Performance per Coronary Segment

When comparing CCTA and ICA results, diagnostic performance varied across coronary segments. Diagnostic indices for all segments are presented in Table 3. In RCA_prox and LAD_prox, sensitivity ranged from 65.8% to 77.3%, while specificity exceeded 70%. In the distal segments and IM, sensitivity was below 40%, but accuracy and specificity remained above 84%. Across all segments, the negative predictive value (NPV) exceeded 80%, while the positive predictive value (PPV) was below 45%.

### 3.3. Artery-Level Diagnostic Analysis

#### 3.3.1. Diagnostic Performance of CCTA Compared with ICA by Artery-Level

Evaluating the diagnostic performance of CCTA by main coronary arteries (Table 4), we observed that sensitivity was highest in LAD, lower in RCA, and lowest in LCX. The positive predictive value (PPV) was limited across all arteries (42.9–50.0%), whereas the negative predictive value (NPV) remained consistently high, ranging from 75.8% to 89.5%.

#### 3.3.2. ROC Curve Analysis of Coronary Arteries

**Figure 1:**
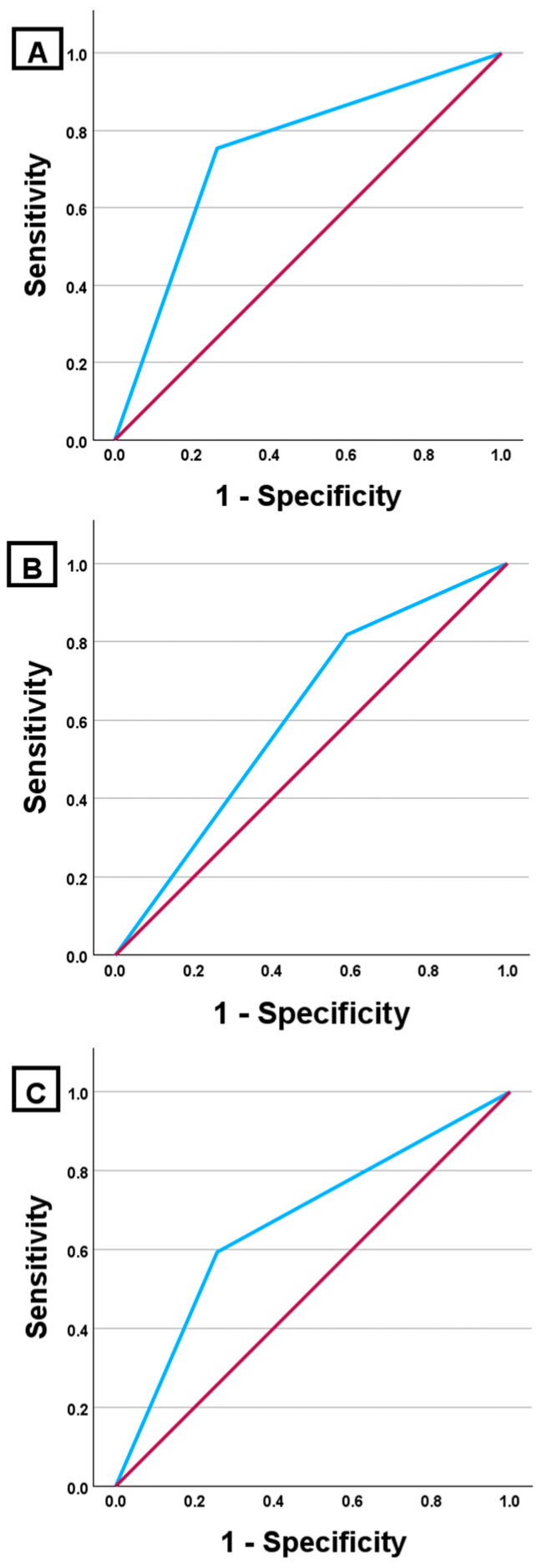
Receiver operating characteristic (ROC) curves demonstrating the diagnostic performance of CCTA compared with ICA for detection of ≥50% coronary stenosis at the artery level. (**A**) RCA, AUC = 0.744 (95% CI 0.694–0.795); (**B**) LAD, AUC = 0.614 (95% CI 0.564–0.663); (**C**) LCX, AUC = 0.668 (95% CI 0.611–0.725). ICA served as the reference standard. The blue line represents the diagnostic performance of CCTA, while the red diagonal line represents the reference line indicating no discriminative ability (AUC = 0.5).

The highest diagnostic accuracy was observed in the right coronary artery (RCA), with an AUC of 0.744 (95% CI 0.694–0.795; *p* < 0.001). In contrast, the left circumflex artery (LCX) and the left anterior descending artery (LAD) demonstrated limited discriminative ability, with an AUC of 0.668 (95% CI 0.611–0.725; *p* < 0.001) and 0.614 (95% CI 0.564–0.663; *p* < 0.001), respectively (Figure 1).

### 3.4. Patient-Level Analysis

At the patient level, CCTA identified at least one coronary segment with ≥50% stenosis in 431 patients (86.2%), whereas ICA identified ≥50% stenosis in 297 patients (59.4%). Concordant findings between the two methods were observed in 314 patients (62.8%), including <50% stenosis in both modalities in 43 patients (8.6%) and ≥50% stenosis in both modalities in 271 patients (54.2%). Among patients with <50% stenosis on CCTA, 26 (5.2%) had ≥50% stenosis on ICA; of these, 7 underwent percutaneous coronary intervention (PCI), whereas the remaining 19 (3.8%) received optimal medical therapy. All percentages are calculated relative to the total study population. The difference between CCTA and ICA was statistically significant (McNemar test, *p* < 0.001). The calculated κ coefficient indicated weak agreement between the two methods. At the patient level, the diagnostic performance of CCTA for detecting at least one ≥50% coronary stenosis across all segments was summarised in Table 5.

### 3.5. Functional and Interventional Outcome Analysis

#### 3.5.1. Relationship Between Structural (CCTA) and Functional (iFR) Assessment

Among all 500 patients, CCTA detected ≥50% stenosis in 86.2% (431/500). A functional evaluation (iFR) during ICA was performed in 6.2% (31/500) of patients, and in most of these cases, CCTA had shown ≥50% stenosis (96.8%; 30/31). A positive iFR result during ICA was observed in 22.6% of patients who underwent iFR evaluation (7/31; 95% CI 11.4–39.8) and in 1.4% of the total study population (7/500). CCTA-detected ≥50% stenosis was associated with a higher likelihood of undergoing iFR assessment (*p* = 0.040). Among patients with CCTA ≥50% stenosis who underwent functional assessment, 76.7% (23/30) had functionally insignificant lesions.

#### 3.5.2. Association Between CCTA Findings and Invasive Treatment

In 431 patients, CCTA detected at least one significant coronary artery stenosis (≥50%); however, PCI was performed in only 140 patients (32.5%). Among 69 patients in whom CCTA did not show significant coronary stenosis, PCI was still performed in 7 cases (10.1%). A statistically significant association was observed between CCTA findings and the frequency of invasive treatment (χ^2^ = 14.30; *p* < 0.001).

**Figure 2:**
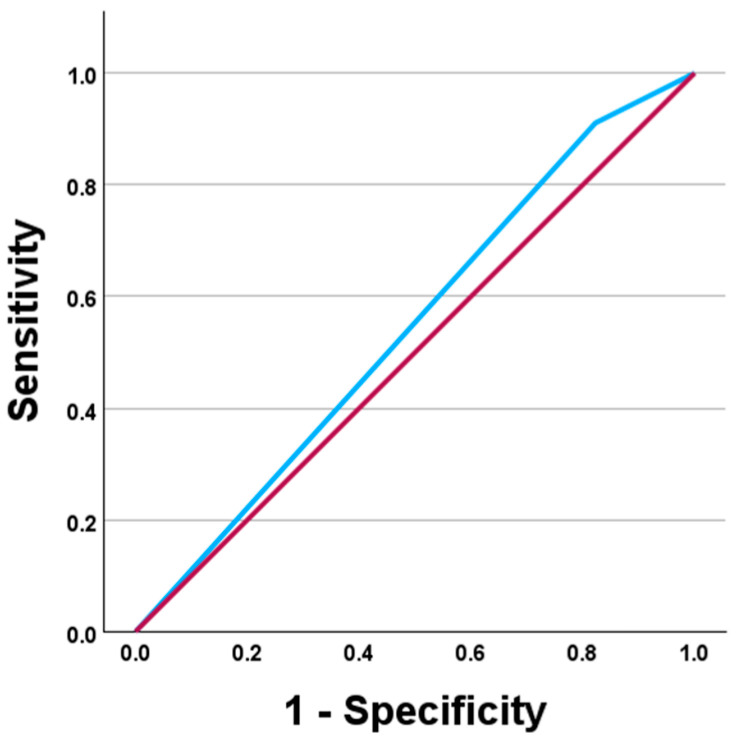
Receiver operating characteristic (ROC) curve demonstrating the ability of ≥50% stenosis on CCTA to discriminate patients who underwent invasive treatment (PCI or CABG) at the patient level (*n* = 500). AUC = 0.544 (95% CI 0.494–0.595). The blue line represents the diagnostic performance of CCTA, while the red diagonal line represents the reference line indicating no discriminative ability (AUC = 0.5).

ROC analysis demonstrated limited ability of the ≥50% stenosis criterion on CCTA to discriminate patients who underwent invasive treatment (AUC = 0.544; 95% CI 0.494–0.595; *p* = 0.086). At this threshold, sensitivity was high (95.2%), whereas specificity was low (17.6%) (Figure 2).

### 3.6. Diagnostic Value of Myocardial Perfusion Scintigraphy Compared with Invasive Coronary Angiography

Myocardial perfusion scintigraphy was performed in 56 patients (11.2% of the total study population). A pathological result was found in 37 patients (66.1%), while 19 patients (33.9%) had normal findings. Perfusion defects were most often observed in the area supplied by the right coronary artery (RCA), found in 28 patients (50.0%). Less frequently, defects were found in the territories of the left circumflex artery (LCX), 12 patients (21.4%), and the left anterior descending artery (LAD), 5 patients (8.9%).

At the patient level, pathological myocardial perfusion scintigraphy results were more frequent among patients in whom invasive coronary angiography (ICA) revealed at least one significant coronary artery stenosis (≥50%) (78.3% vs. 26.3%), although this difference was not statistically significant (*p* = 0.108). The diagnostic performance of myocardial perfusion scintigraphy compared with ICA showed a sensitivity of 78.3% and a specificity of 42.4%, with low overall agreement between the two methods (κ = 0.189 ± 0.221). No significant associations were observed between perfusion defect localisation and corresponding ICA findings in RCA, LAD, and LCX territories. Quantitative perfusion values (rest, stress, and their difference) also did not differ significantly between groups (*p* > 0.05).

### 3.7. Factors Associated with Discrepancies Between CCTA and ICA Results

#### 3.7.1. Vessel-Level Analysis: Association Between Agatston Score

Men demonstrated significantly higher Agatston scores across all analysed coronary arteries compared with women. The differences were statistically significant for the total Agatston score (*p* = 0.012), as well as for the right coronary artery (*p* = 0.040), left anterior descending artery (*p* = 0.004), left circumflex artery (*p* = 0.005), and left main artery (*p* = 0.006).

In RCA, the total Agatston score was statistically significantly higher in the discordant group (*p* = 0.029). The RCA-localised calcification index was also higher, although the difference did not reach statistical significance (*p* = 0.055) (Table 6).

In LAD, the total Agatston score was statistically significantly higher in the discordant group (*p* = 0.037). The LAD-localised Agatston score was also statistically significantly higher in the discordant group (*p* = 0.011) (Table 7).

In LCX, a statistically significant association was found only for the LCX-localised calcification index (*p* = 0.015). The total Agatston score was not statistically significant (*p* = 0.207) (Table 8).

In the LM region, the discordant group showed a significantly higher Agatston score in the LM itself (*p* = 0.036), as well as in the adjacent LAD (*p* = 0.018) and LCX (*p* = 0.015) segments. The total calcium burden was also higher in this group, although the difference did not reach statistical significance (*p* = 0.081) (Table 9).

#### 3.7.2. Patient-Level Analysis: Influence of Demographic, Anthropometric, Cardiac and Comorbid Factors

No statistically significant associations were found between CCTA–ICA concordance and the analysed clinical factors. However, a trend toward higher discrepancy rates was observed among patients with diabetes mellitus and cerebrovascular atherosclerosis, although these differences did not reach statistical significance (*p* = 0.072 and *p* = 0.085, respectively) (Table 10).

Patients with discordant CCTA and ICA findings had a significantly higher BMI, whereas age and sex were not significantly associated with discrepancies (Table 11).

#### 3.7.3. Patient-Level Analysis: Multivariate Analysis of Factors Associated with CCTA–ICA Discrepancies

In multivariate analysis, higher BMI (>25 kg/m^2^), younger age (<68 years), and the presence of more than two comorbidities remained independently associated with CCTA–ICA discrepancies. The model demonstrated a modest overall classification accuracy of 63.1%, and the statistical significance of the predictors ranged from *p* = 0.015 to *p* = 0.028 (Table 12).

### 3.8. Evaluation of Coronary Circulation Dominance Based on CCTA and ICA

The type of coronary circulation dominance determined by CCTA and ICA coincided in 84.8% of cases. Agreement between the two methods was moderate, with a linear weighted kappa of 0.454 (95% CI 0.350–0.559) and a quadratic weighted kappa of 0.413 (95% CI 0.294–0.532). Among concordant cases (*n* = 357), right-dominant circulation was observed in 320 patients (89.6%). Among discordant cases (*n* = 64), discrepancies most frequently involved disagreement between right-dominant and balanced circulation, accounting for 40 cases (62.5%).

## 4. Discussion

This study evaluated the concordance and diagnostic performance of CCTA in a clinically referred cohort undergoing ICA, with analyses performed at the patient, vessel, and segment levels. The cohort comprised 500 patients with a mean age of 67.3 ± 9.4 years, reflecting an older, clinically higher-risk population in whom advancing age is an established independent risk factor for atherosclerotic cardiovascular disease [5,6,7]. Women were significantly older than men, consistent with epidemiological data indicating that CAD typically manifests later in women, partly due to the cardioprotective effects of oestrogen before menopause and their decline thereafter [8,9]. Compared with major CCTA cohorts, our population was slightly older, likely due to clinical selection, as older patients more often present with cardiovascular risk factors and symptoms requiring CCTA or ICA [10,11,12]. As expected, nearly all participants had at least one comorbidity. Arterial hypertension was highly prevalent, and left ventricular dysfunction was also common, in line with previously reported CCTA cohorts [13,14].

Beyond patient characteristics, concordance between CCTA and ICA demonstrated significant variability across coronary segments. At the segment level, weighted κ analysis showed poor-to-moderate agreement, consistent with previous studies reporting only moderate concordance in anatomical stenosis grading despite strong diagnostic performance at the patient level [15]. Our analysis revealed marked segment-specific differences, with the highest agreement observed in the proximal RCA and the lowest concordance in the distal LCX, intermediate branches, and the left main coronary artery. Importantly, agreement did not uniformly decline from proximal-to-distal segments across all vessels. In the RCA, distal segments demonstrated higher κ values than mid segments, whereas in the LAD, agreement was greater in the mid segment than in the proximal segment. These findings likely reflect segment-specific disease prevalence, referral bias inherent to an ICA-based cohort, and the sensitivity of κ statistics to unbalanced category distributions. They are less likely to represent true differences in diagnostic capability. Similar segment-dependent discordance between CCTA and ICA has been reported in contemporary comparative studies, particularly involving distal vessels, the LCX, and intermediate branches. For example, Pagonis et al. reported that CCTA detected only 44% of lesions identified by ICA in distal coronary segments [15,16]. This segment-specific heterogeneity likely reflects underlying anatomical complexity, including smaller vessel diameter, increased tortuosity, bifurcation geometry, vessel dominance patterns, and a higher prevalence of calcification [17,18]. Notably, no statistically significant differences between CCTA- and ICA-derived stenosis classifications were observed in the distal RCA and intermediate branches, suggesting that in selected segments CCTA may provide anatomically comparable assessments to invasive angiography. Thus, despite high patient-level diagnostic accuracy, segment-level variability remains clinically relevant. These findings emphasise the importance of standardised acquisition and interpretation protocols and support cautious interpretation of stenosis severity in distal and left-sided coronary segments.

Analysis of segment-level diagnostic performance further demonstrated that CCTA maintains high sensitivity and NPV for detecting significant coronary artery stenosis, particularly in proximal segments, whereas specificity and PPV varied across the coronary tree and were lower in distal segments. Recent segment-based studies have reported that the sensitivity of CCTA for detecting ≥50% stenosis ranges from approximately 58% to 87.5%, with consistently high NPV, while specificity and PPV vary according to coronary calcium burden and disease prevalence [19,20]. In our cohort, NPV remained high across all coronary segments (generally >80%), whereas sensitivity showed substantial variability and was markedly lower in distal segments, in some cases below 40%, with corresponding fluctuations in specificity and PPV. This variability may partly reflect the known tendency of CCTA to overestimate stenosis severity compared with ICA, potentially leading to higher detection rates and risk reclassification, particularly in segments where ICA assessment may underestimate non-obstructive disease [21]. Overall, these findings support current guideline recommendations, reinforcing the role of CCTA as a reliable rule-out modality for significant coronary artery disease, especially in proximal and mid segments, while underscoring the need for careful interpretation and selective ICA in distal or heavily calcified vessels.

When diagnostic performance was evaluated at the vessel level, marked heterogeneity was observed across the major coronary territories, likely reflecting differences in anatomical configuration and image quality. The LAD is generally easier to visualise due to its larger proximal diameter, more anterior position, and relatively straighter course, whereas the LCX often follows a more tortuous path within the atrioventricular groove and may be deeper or partially obscured by adjacent cardiac structures, potentially reducing image quality and sensitivity [22,23,24,25,26,27]. In the ACCURACY trial, vessel-based analysis across the major coronary arteries demonstrated a sensitivity of 84% and specificity of 90% for detecting ≥50% stenosis [10]. Similar findings were reported by Herzog et al., who observed high per-vessel diagnostic performance (sensitivity 88.8%, specificity 91.5%) without significant differences among RCA, LAD, and LCX territories [28]. In contrast, our artery-specific analysis revealed more pronounced heterogeneity, with higher sensitivity but lower specificity in the LAD and the opposite pattern in the LCX. Because coronary territories are defined differently across studies, direct numerical comparisons should be interpreted cautiously; therefore, we focus on overall diagnostic patterns rather than exact percentage values. Consistent with the typical CCTA diagnostic profile, NPV remained high across all three arteries (75.8–89.5%), whereas PPV was limited (42.9–50.0%). Clinically, this supports the role of CCTA as a reliable rule-out modality for significant coronary stenosis. The relatively low PPV may be explained by overestimation of stenosis severity due to blooming artifacts from heavily calcified plaques, as well as by smaller vessel diameter and increased tortuosity [29,30,31,32]. This interpretation is supported by image-processing approaches, as de-blooming CCTA algorithms improve the assessment of calcified plaques and increase specificity and PPV, indicating that calcification substantially contributes to false-positive findings [33]. Finally, PPV and NPV are inherently population- and methodology-dependent metrics. They vary according to pretest probability, disease prevalence, stenosis thresholds, level of analysis (per-patient versus per-vessel), handling of non-diagnostic segments, and overall image quality [34,35,36,37].

Further insight into artery-specific diagnostic performance was obtained through ROC curve analysis, which demonstrated heterogeneous discriminatory ability across the major coronary territories. In our cohort, ≥50% stenoses were most accurately differentiated within the RCA territory. The relatively higher discriminatory performance observed in the RCA may reflect vessel-specific differences in CCTA accessibility and image quality. Previous studies have reported superior segment-level image quality in the RCA, whereas the LCX is more frequently associated with suboptimal visualisation. Reduced image quality—particularly in the presence of extensive calcification—may contribute to stenosis misclassification and thereby influence discriminatory performance metrics [31,34].

In contrast to the pronounced variability observed at the segment and artery levels, patient-level analysis demonstrated a more consistent diagnostic profile of CCTA. The findings showed high CCTA sensitivity and NPV for the detection of at least one coronary ≥50% stenosis, whereas specificity and PPV were more limited when compared with ICA. In the present cohort, patient-level agreement between CCTA and ICA was modest, reflecting a systematic tendency of CCTA to classify a larger proportion of patients as having at least one anatomically significant stenosis. This pattern is consistent with the established diagnostic profile of CCTA, in which prioritisation of sensitivity comes at the expense of specificity. Large multi-centre studies have similarly reported high patient-level sensitivity for CCTA, typically ranging from 85% to 96%, alongside more variable specificity between 72% and 90%, with consistently high NPV and moderately high PPV [38,39]. The Society of Cardiovascular Computed Tomography 2021 Expert Consensus Document emphasises this trade-off, highlighting the role of CCTA in excluding anatomically significant CAD [40]. In this context, the limited patient-level agreement observed in our study likely reflects differences in diagnostic thresholds rather than true discordance, underscoring that while CCTA is effective as a rule-out test, ICA remains the reference standard for definitive anatomical characterisation in patients with suspected or complex CAD.

The relationship between structural and functional assessment further underscores the limitations of anatomy-based decision-making. Our data showed that even in the presence of ≥50% stenosis, a substantial proportion of lesions were not hemodynamically significant by iFR; therefore, decisions regarding revascularisation based on anatomical criteria alone may be insufficient. Among patients in whom CCTA showed ≥50% stenosis and iFR was performed, 76.7% of lesions were functionally insignificant and interventional treatment was not indicated. This highlights the importance of physiological confirmation and is consistent with the evidence base supporting invasive physiology-guided strategies [41,42,43]. However, iFR was performed in only 6.2% of participants; therefore, the results of this subgroup may not reflect the entire cohort. It is likely that iFR was used more often in cases of intermediate stenosis or when there was clinical uncertainty, which could have increased the proportion of functionally insignificant lesions in this subgroup. Although functional assessment in our centre was performed invasively during ICA, current practice also allows functional significance to be assessed non-invasively from CCTA data using CT-FFR, which, compared with anatomical CCTA assessment alone, increases specificity and diagnostic discrimination and reduces the likelihood of unnecessary invasive coronary angiography [44,45]. Given that most anatomically significant stenoses in our iFR-assessed subgroup were functionally insignificant, adding non-invasive functional assessment (CT-FFR) to the CCTA pathway could reduce unnecessary invasive procedures and potentially avoid overtreatment [46].

Similarly, the predictive value of CCTA for invasive treatment was limited when anatomical thresholds alone were applied. Although a statistically significant association was found between CCTA results and the frequency of invasive treatment (χ^2^ = 14.30; *p* < 0.001), the CCTA criterion of “≥50% stenosis in at least one segment” had limited predictive value for invasive treatment (PCI or CABG) (AUC = 0.544; 95% CI 0.494–0.595; *p* = 0.086). This is reflected by very high sensitivity (95.2%) combined with extremely low specificity (17.6%), indicating that an anatomical ≥50% threshold identifies more patients as potential candidates for invasive treatment, although many do not ultimately undergo revascularisation. As an anatomy-only test, CCTA does not determine whether stenosis is haemodynamically significant; therefore, a ≥50% anatomical threshold alone is expected to have low specificity for predicting revascularisation [47]. The decision to revascularise is often based on physiological assessment, so anatomy alone is not sufficient to determine the need for intervention [41]. In the NXT study, CT-FFR significantly increased specificity compared to CCTA anatomical assessment alone, highlighting the limitations of anatomy-based thresholds for patient selection [45]. A large-scale analysis confirmed that a substantial proportion of stenoses show discordance between angiographic and FFR criteria, especially in moderate lesions, which may explain why some patients with ≥50% stenosis on CCTA do not undergo revascularisation [48]. ESC guidelines recommend integrating anatomical information with functional assessment, especially for intermediate grade stenoses [49]. Thus, our findings support the concept that anatomical information should be evaluated along with functional assessment to reduce the overestimation of the need for invasive revascularisation.

In addition to anatomical and physiological coronary assessment, evaluation of MPS revealed limited ability to reliably identify anatomically defined coronary stenoses. The findings were less favourable than those reported in larger meta-analyses, which describe pooled sensitivity of approximately 83–88% and specificity of 61–77% when ICA is used as the reference standard [50,51]. The discrepancy may partly reflect the small sample size and patient selection in the present study, as well as the well-recognised mismatch between functional perfusion abnormalities and anatomical stenosis severity, as evidenced by the absence of a significant association between perfusion defect localisation and angiographic findings. This phenomenon is described in patients with multivessel disease, diffuse atherosclerosis, or balanced ischemia, where MPS may underestimate lesion-specific ischemia despite angiographically significant disease [52,53]. This imperfect correlation reflects the known inherent discordance between functional ischemia assessment and purely anatomical stenosis severity, particularly when invasive coronary angiography is used as the sole reference standard. Large, randomised trials, including DEFINE-FLAIR and iFR-SWEDEHEART, demonstrated that physiological indices such as FFR and iFR are superior to angiography-based stenosis severity for guiding revascularisation decisions, with safe deferral of intervention and comparable rates of major adverse cardiac events at both short and long-term follow-up [54,55,56]. These findings reinforce the concept that functional and anatomical assessments provide complementary but non-equivalent information, and that reliance on MPS alone may be insufficient for precise lesion-level or vessel-specific decision-making when ICA is used as the sole reference standard.

Beyond the functional–anatomical mismatch, image quality-related factors also play an important role in explaining discordance between non-invasive and invasive coronary assessments. Coronary calcification burden emerged as an important determinant of CCTA-ICA discordance, with artery-specific differences and distinct effects of local versus total Agatston scores. High calcification burden is a known limiting factor of CCTA diagnostic performance; the CORE-64 analysis showed that a high Agatston score reduces overall CCTA accuracy and discriminatory ability [31,35]. In our study, men had a higher Agatston score (both total and per artery), consistent with population cohort data showing higher Agatston scores in men, and this may contribute to more frequent CCTA-ICA discordance in the male subgroup [32,57]. Mechanistically, calcification can cause blooming/partial-volume artifacts on CCTA and complicates the lumen/stenosis assessment, thereby increasing the likelihood of discordance with ICA [58,59,60,61]. Our results demonstrate that the relationship between the Agatston score and CCTA–ICA discordance is artery-dependent and differs according to whether coronary artery calcium burden is assessed locally or globally. This pattern is consistent with prior studies showing that increased calcification burden leads to an increase in false-positive stenosis diagnoses on CCTA, thereby lowering specificity and increasing the risk of CCTA-ICA discordance [31,32,60]. The results are limited by small discordant subgroup sizes, multiple comparisons, and unassessed direction of discordance (false positive vs. false negative), and should therefore be interpreted with caution. Clinically, these findings underscore the need for more cautious interpretation of CCTA stenosis assessments in patients with a high Agatston score and for additional verification when uncertainty remains.

Patient-level characteristics were also evaluated to assess their potential contribution to diagnostic disagreement; however, no statistically significant associations were found between CCTA-ICA discordance and the clinical or demographic factors evaluated (all *p* > 0.05). BMI was higher in the discordant group, and patients with a BMI ≥ 25 kg/m^2^ had almost twofold higher odds of discordance (OR 1.97; 95% CI 1.15–3.39). The association between higher BMI and discordance may be explained by technical factors: as body mass increases, photon attenuation and scattering increase, leading to higher image noise, which makes it more difficult to assess the artery lumen and plaque margins and increases uncertainty in stenosis assessment [62]. Alkadhi et al. showed that a high BMI was associated with lower per-patient specificity compared to ICA, while maintaining high sensitivity and NPV, consistent with discordance driven by false-positive (overestimated) findings [63]. From a practical perspective, this indicates that CCTA interpretation may be less accurate in patients with higher BMI; therefore, ensuring optimal CCTA image quality and protocol optimisation is important. Although none of the comorbidities evaluated were statistically significantly associated with CCTA-ICA discordance, non-significant trends were observed in patients with diabetes mellitus or cerebrovascular atherosclerosis (*p* = 0.072 and *p* = 0.085, respectively). These results should be interpreted with caution, as the 95% CI included 1. Age and sex were also not statistically significantly associated with CCTA-ICA discordance; therefore, patient-level discordance is better explained by technical/image quality factors than by demographic characteristics or cardiac and comorbid factors.

To further clarify the independent contribution of patient-related factors, a multivariate logistic regression analysis was performed. Multivariable logistic regression analysis identified several independent factors associated with CCTA–ICA discordance, including BMI ≥ 25 kg/m^2^, age < 68 years, and the presence of more than two comorbidities. However, the overall discriminatory performance of the model was modest, indicating limited classification accuracy. Therefore, these findings should be interpreted cautiously and considered hypothesis-generating rather than definitive predictors of inter-modality mismatch. The observed associations may reflect a cumulative effect of technical and atherosclerotic phenotype factors rather than a single isolated determinant. Overweight was the strongest predictor, as higher BMI degrades CCTA image quality, mainly due to technical and image quality barriers, and increases stenosis classification error [62]. The association with younger age likely reflects a higher prevalence of non-calcified/mixed plaques and their positive remodelling, increasing the proportion of borderline stenoses and variability in CCTA interpretation [64,65,66]. A higher multimorbidity burden may act as a marker of diffuse and more frequently calcified atherosclerosis; calcification reduces the accuracy of CCTA due to blooming/partial-volume artifacts and lower specificity [31,67,68]. Clinically, these findings support identifying higher-risk subgroups and emphasise CCTA protocol optimisation and image quality control.

Finally, assessment of coronary circulation dominance demonstrated good overall agreement between CCTA and ICA. In this study, the classification of coronary circulation dominance using CCTA and ICA matched in 84.8% of cases, with κ analysis indicating moderate concordance between the two modalities. Right-dominant circulation was the most prevalent pattern, while most inconsistencies arose in cases of balanced dominance. These results align with prior studies demonstrating that CCTA can accurately assess coronary dominance, particularly right-dominant circulation, which has been reported in over 90% of patients in large cohorts [69,70]. Barbieri et al. indicate that agreement may vary depending on technical, interpretative, and clinical factors, with lower overall concordance observed in some settings and differences across patient subgroups rather than systematic misclassification [71]. Within this context, the moderate kappa values observed in the present study are comparable to, or slightly higher than, those reported previously. Overall, these results support the use of CCTA as a reliable non-invasive modality for coronary dominance assessment, with discrepancies largely confined to equivocal anatomical classifications, while ICA remains the reference standard in complex cases.

This study has several limitations that should be considered when interpreting the findings. It was a single-centre retrospective analysis and is therefore subject to the inherent limitations of this design, including potential selection bias and incomplete control of confounding factors. As only patients referred for invasive coronary angiography were included, referral and verification bias cannot be excluded and may have influenced the observed prevalence of ≥50% stenosis as well as diagnostic performance estimates. Consequently, the study population represents a clinically higher-risk cohort rather than an unselected CCTA population. The interval of up to one year between CCTA and ICA may have allowed for potential progression or modification of coronary atherosclerosis, which could have influenced inter-modality concordance. Invasive physiological assessment using iFR was available only in a limited subset of patients and was not systematically performed across the cohort. Although Agatston score was evaluated in artery-level analyses, it was not incorporated into the final patient-level multivariable model due to incomplete availability, which may have limited adjustment for coronary calcification burden. In addition, multiple comparisons were performed, particularly in analyses involving Agatston score across different coronary territories, without formal correction for multiple testing; therefore, these findings should be interpreted cautiously and considered exploratory. Furthermore, the direction of CCTA–ICA discordance (false-positive versus false-negative findings) was not analysed separately, limiting more detailed mechanistic interpretation of the associations observed with coronary calcification burden and BMI. Finally, although multivariable modelling identified independent predictors of CCTA–ICA discordance, the overall discriminatory performance of the model was modest, suggesting that additional unmeasured technical, anatomical, or clinical factors may contribute to inter-modality mismatch.

## 5. Conclusions

CCTA is an effective non-invasive tool for ruling out significant coronary artery disease at the patient level. However, its diagnostic agreement with ICA remains limited at the segmental and vessel levels, particularly in distal segments and left-sided coronary arteries. Although CCTA frequently identified coronary stenosis ≥50%, this anatomical finding alone was not sufficient to guide decisions regarding invasive treatment. A substantial proportion of anatomically significant stenoses were functionally insignificant, highlighting the risk of overestimation when relying solely on anatomical assessment. Discrepancies between CCTA and ICA are strongly influenced by technical and anatomical factors, including coronary calcification burden and increased BMI, which reduce image interpretability and specificity. These findings support the role of CCTA as a non-invasive diagnostic modality, while decisions regarding invasive management should rely on integrated anatomical and functional assessment, interpreted within the overall clinical context. Further prospective multi-centre studies with more robust methodological designs are warranted to confirm these findings and to refine the clinical role of CCTA in patient selection for invasive evaluation.

## Figures and Tables

**Table 1:** Distribution of comorbidities in the study population (*n* = 500). Values are presented as number (%) of patients. Multiple comorbidities per patient were allowed.

Diagnosed Condition	*n* (%)
Arterial hypertension	474 (94.8)
Left ventricular failure	398 (79.6)
Rhythm or conduction disorders	109 (21.8)
Diabetes mellitus	85 (17.0)
Atrial fibrillation	68 (13.6)
Heart failure	50 (10.0)
Chronic obstructive pulmonary disease	32 (6.4)
Cerebrovascular atherosclerosis	22 (4.4)
Implanted pacemaker	17 (3.4)
Peripheral arterial disease	16 (3.2)

**Table 2:** Segment-level agreement between CCTA and ICA stenosis assessment based on weighted kappa coefficients. Values are presented as κ ± standard error (SE). Agreement was evaluated using both six-grade and binary (<50% vs. ≥50%) stenosis classifications at the segment level.

Segment	Weighted ^1^ κ ± SE ^2^ (6-Grade ^3^)	κ ± SE (2-Grade ^4^)
RCA_prox	0.436 ± 0.053	0.461 ± 0.043
RCA_mid	0.290 ± 0.064	0.232 ± 0.069
RCA_dist	0.356 ± 0.084	0.306 ± 0.080
LM	0.113 ± 0.042	0.149 ± 0.104
LAD_prox	0.295 ± 0.042	0.241 ± 0.042
LAD_mid	0.386 ± 0.046	0.254 ± 0.040
LAD_dist	0.257 ± 0.067	0.188 ± 0.059
IM	0.138 ± 0.080	0.132 ± 0.084
LCX_prox	0.289 ± 0.059	0.267 ± 0.059
LCX_mid	0.267 ± 0.054	0.201 ± 0.051
LCX_dist	0.108 ± 0.054	0.110 ± 0.061

^1^ Quadratic weighted kappa (κ); ^2^ SE—standard error; ^3^ 6-grade classification: 0%, <50%, 50–69%, 70–89%, 90–99%, 100%; ^4^ 2-grade classification: <50% and ≥50%.

**Table 3:** Segment-level diagnostic performance of CCTA compared with ICA using the ≥50% stenosis threshold. Values are presented as percentages. ICA served as the reference standard. PPV—positive predictive value; NPV—negative predictive value.

Segment	Sensitivity (%)	Specificity (%)	Accuracy (%)	PPV (%)	NPV (%)
RCA_prox	77.3	79.7	79.2	38.0	95.1
RCA_mid	35.3	92.3	88.4	25.0	95.1
RCA_dist	37.9	95.1	91.8	32.4	96.1
LM	25.0	97.2	96.0	12.5	98.8
LAD_prox	65.8	71.0	70.2	28.9	92.1
LAD_mid	69.9	59.6	62.8	44.0	81.4
LAD_dist	35.7	88.4	84.0	22.1	93.8
IM	21.4	95.9	93.8	13.0	97.7
LCX_prox	52.6	87.2	84.6	25.3	95.7
LCX_mid	42.7	82.2	76.8	27.4	90.1
LCX_dist	12.5	96.0	88.0	25.0	91.2

**Table 4:** Artery-level diagnostic performance of CCTA compared with ICA using the ≥50% stenosis threshold. Values are presented as percentages. ICA served as the reference standard. PPV—positive predictive value; NPV—negative predictive value.

Artery	Sensitivity (%)	Specificity (%)	PPV (%)	NPV (%)
RCA	75.4	73.5	50.0	89.5
LAD	81.8	40.9	49.9	75.8
LCX	59.4	74.3	42.9	84.9

**Table 5:** Patient-level diagnostic performance of CCTA compared with ICA for detection of at least one ≥50% coronary stenosis across all 11 segments. Values are presented as percentages. ICA served as the reference standard. PPV—positive predictive value; NPV—negative predictive value.

Indicator	Value (%)
Sensitivity	91.3
Specificity	21.2
Accuracy	62.8
PPV	62.9
NPV	62.3

**Table 6:** Association between Agatston score and CCTA–ICA discrepancies in RCA. Values are presented as the median (IQR) and number of patients.

Parameter	Discordant	Concordant	*p*-Value
Total Agatston score	1070 (330–1620) (*n* = 10) ^1^	244 (61–288) (*n* = 48)	0.029
RCA Agatston score	291 (107–615) (*n* = 10) ^1^	35 (0–106) (*n* = 40)	0.055

^1^ The discordant subgroup was small (*n* = 10); therefore, the findings should be interpreted with caution.

**Table 7:** Association between Agatston score and CCTA–ICA discrepancies in LAD. Values are presented as the median (IQR) and number of patients ^1^.

Parameter	Discordant	Concordant	*p*-Value
Total Agatston score	451 (152–1255) (*n* = 31)	92 (19–1167) (*n* = 27)	0.037
LAD Agatston score	210 (90–583.25) (*n* = 28)	62 (17–312) (*n* = 21)	0.011

^1^ Subgroup sizes differed between groups; therefore, the results should be interpreted cautiously.

**Table 8:** Association between Agatston score and CCTA–ICA discrepancies in LCX. Values are presented as the median (IQR) and number of patients.

Parameter	Discordant	Concordant	*p*-Value
Total Agatston score	714 (110–1411) (*n* = 15)	285 (64–765) (*n* = 43)	0.207
LCX Agatston score	166 (35.75–321) (*n* = 12) ^1^	13.5 (0–106.5) (*n* = 36)	0.015

^1^ The discordant subgroup was relatively small (*n* = 12); therefore, the findings should be interpreted with caution.

**Table 9:** Association between Agatston score and CCTA–ICA discrepancies in LM. Values are presented as the median (IQR) and number of patients.

Parameter	Discordant	Concordant	*p*-Value
Total Agatston score	1255 (539–1335) (*n* = 5) ^1^	285 (62–825) (*n* = 53)	0.207
LM Agatston score	36 (18.5–274.5) (*n* = 5) ^1^	0 (0–50.75) (*n* = 44)	0.015
LAD Agatston score	356 (329–747.5) (*n* = 5) ^1^	116.5 (33.75–316.75) (*n* = 44)	0.018
LCX Agatston score	239 (112–301) (*n* = 5) ^1^	19 (0–110) (*n* = 43)	0.015

^1^ The discordant subgroup was very small (*n* = 5); therefore, findings should be interpreted with caution.

**Table 10:** Cardiologic and comorbid conditions associated with concordance between CCTA and ICA at the patient level. Values are presented as number (%). Odds ratios (OR) with 95% confidence intervals (CI) are shown for each condition. Concordant and discordant groups were defined according to agreement between CCTA and ICA for ≥50% stenosis.

Condition	Concordance If Absent *n* (%)	Concordance If Present *n* (%)	*p*-Value	OR (95% CI)
Heart failure	287 (63.8)	27 (54.0)	0.175	0.667 (0.37–1.20)
Left ventricular failure	63 (61.8)	251 (63.1)	0.808	1.057 (0.68–1.66)
Rhythm or conduction disorders	248 (63.6)	66 (60.6)	0.561	0.879 (0.57–1.36)
Atrial fibrillation	276 (64.0)	38 (55.9)	0.196	0.711 (0.42–1.19)
Arterial hypertension	17 (68.0)	296 (62.4)	0.576	0.783 (0.33–1.85)
Diabetes mellitus	267 (64.5)	46 (54.1)	0.072	0.649 (0.41–1.04)
Peripheral artery disease	306 (63.2)	8 (50.0)	0.282	0.582 (0.22–1.58)
Cerebrovascular atherosclerosis	304 (63.6)	10 (45.5)	0.085	0.477 (0.20–1.13)
Chronic obstructive pulmonary disease	292 (62.4)	22 (68.8)	0.472	1.326 (0.61–2.87)
Implanted pacemaker	301 (62.3)	13 (76.5)	0.311	1.965 (0.631–6.118)

**Table 11:** Association between demographic factors and CCTA–ICA discrepancies at the patient level. Values are presented as the median (IQR) or number (%). Concordant and discordant groups were defined according to agreement between CCTA and ICA for ≥50% stenosis.

Factor	Concordant	Discordant	*p*-Value
Age (years), median [IQR]	69 (62–74)	66 (60–73)	0.061
Sex (men/women)	164 (52.2%)/150 (47.8%)	85 (45.7%)/101 (54.3%)	0.173
BMI (kg/m^2^)	28.76 (25.36–32.27)	30.15 (26.73–33.67)	0.024

**Table 12:** Multivariable binary logistic regression model for prediction of CCTA–ICA discrepancy at the patient level. Variables with *p* < 0.05 in univariable analysis were included in the multivariable model. Results are presented as odds ratios (OR) with 95% confidence intervals (CI).

Predictor	Univariate OR ^1^ (95% CI ^2^)	B Coefficient ^3^	Multivariate OR ^1^ (95% CI ^2^)	*p*-Value
BMI ^4^ > 25.0 kg/m^2^	1.974 (1.149–3.389)	0.614	1.848 (1.069–3.196)	0.028
Age < 68 years	1.509 (1.146–2.176)	0.504	1.655 (1.102–2.483)	0.015
>2 comorbidities	1.450 (1.001–2.104)	0.490	1.632 (1.081–2.464)	0.020
Constant	–	–1.459	–	<0.001

^1^ Odds ratio; ^2^ confidence interval; ^3^ regression coefficient; ^4^ body mass index.

## Data Availability

The original contributions presented in this study are included in the article. Further inquiries can be directed to the corresponding author.
